# Human bocavirus NP1 antagonizes host type I interferon response through repressing the nuclear transport of STAT1

**DOI:** 10.1080/21505594.2025.2570000

**Published:** 2025-10-15

**Authors:** Minfei Xue, Chenxiao Huang, Xiaohui Jiang, Shimeng Zhou, Yongdong Yan, Chunsheng Dong, Zhengrong Chen, Jianfeng Dai

**Affiliations:** a Jiangsu Key Laboratory of Infection and Immunity, Children’s Hospital of Soochow University, Institutes of Biology and Medical l Sciences, Suzhou Medical College of Soochow University, Suzhou, China; b MOE Key Laboratory of Geriatric Diseases and Immunology, Suzhou Medical College of Soochow University, Suzhou, China; c Department of Respiratory Medicine, Children’s Hospital of Soochow University, Suzhou, China

**Keywords:** HBoV1, NP1, STAT1, KPNA1, antiviral immunity, IFN-I signaling pathway

## Abstract

Human bocavirus 1 (HBoV1), first identified in human nasopharyngeal specimens in 2005, is a relatively recent member of the respiratory virus family and is primarily associated with respiratory tract infections. Despite this, the mechanisms underlying HBoV1 pathogenesis remain poorly understood, primarily due to the lack of suitable cell lines and animal models, as well as the complicating factor of co-infections with other pathogens. In this study, we demonstrate that the non-structural protein 1 (NP1) of HBoV1 antagonizes the type I interferon (IFN-I) signaling pathway. NP1 interacts with the DNA-binding domain (DBD) of signal transducer and activator of transcription 1 (STAT1) and the importing β-binding (IBB) domain of karyopherin subunit alpha-1 (KPNA1), thereby disrupting the formation of the STAT1/KPNA1/KPNB1 complex and subsequently inhibiting STAT1 nuclear translocation, a critical step in IFN-I signaling. Furthermore, we show that HBoV1 NP1 facilitates the replication of influenza A virus (IAV) and respiratory syncytial virus (RSV). These findings suggest a novel mechanism by which bocavirus proteins antagonize the host’s innate immune response and provide new evidence that bocavirus may exacerbate the symptoms of clinical respiratory viral infections.

## Introduction

Lower respiratory tract infection (LRTI) is one of the most prevalent diseases and a leading cause of mortality among children under five years of age worldwide, placing a significant burden on both society and families. Human bocavirus (HBoV) is a relatively recent addition to the respiratory virus family, first discovered within the past 20 years. Its association with respiratory diseases in children has attracted increasing attention. HBoV1, identified in human nasopharyngeal specimens in 2005 by Allander and colleagues [1], is a human parvovirus that causes acute respiratory infections in children. HBoV1 infection can cause asthma, bronchiolitis and pneumonia, typical symptoms include rhinorrhea, fever and cough [2,3]. In the following years, three additional HBoV subtypes – HBoV2, HBoV3, and HBoV4—were identified in human stool specimens [4]. While HBoV1 is primarily associated with respiratory tract infections, HBoV2-4 are more commonly linked to gastrointestinal tract infections. The global prevalence of HBoV respiratory infections is currently estimated at approximately 6.3%, with clinical cases frequently showing a high incidence of co-infection with other viral and bacterial pathogens affecting the respiratory or gastrointestinal systems, such as RSV, IAV, human rhinovirus, adenovirus, norovirus, and rotavirus [5–7]. In respiratory samples, up to 83% of cases involve mixed infections, with RSV being the most common co-infecting pathogen, exhibiting a co-infection rate as high as 89.5% [8,9]. In recent years, an increasing number of reports have highlighted that HBoV causes severe infections, some of which are life-threatening.

HBoV1 is a 20-faced, symmetric virus lacking an envelope and possessing a single-stranded DNA genome approximately 5500 bp in length, with “hairpin” structures at both ends [10]. The HBoV1 genome contains three open reading frames (ORFs), which encode six non-structural proteins (NS1, NS1-70, NS2, NS3, NS4, and NP1) and three structural proteins (VP1, VP2, and VP3). Additionally, the HBoV1 genome can transcribe a long non-coding RNA (lncRNA), called BocaSR, that is 140 bp in length [11]. The NS proteins primarily participate in the expression of non-coding RNA, while NP1 plays a crucial role in the expression of viral capsid proteins and mRNA processing, and directly participates in the replication of the viral DNA at the replication origin (OriR) [12]. VP1 contains a unique region of 90 amino acids (VP1 unique, VP1u), which encodes a sequence with an internal polyadenylate signal [12,13]. The VP1u region, spanning amino acids 11 to 66, exhibits phospholipase A2 (PLA2) activity, which is essential for viral transmission. VP2 serves as the main serological immune target and is a promising candidate for a HBoV1 vaccine [14]. VP3 can assemble virus-like particles (VLPs) that contain neutralizing epitopes and receptor binding sites [15]. BocaSR, located in the nucleus, is the first identified RNA polymerase III-transcribed viral lncRNA in parvoviruses. It regulates the expression of NS1, NS2, NS3, and NP1, but not NS4 [16].

Upon viral invasion, the host initiates a series of innate and adaptive immune responses. Innate immunity acts as the first line of defense against viruses, recognizing pathogen-associated molecular patterns (PAMPs) through host pattern recognition receptors (PRRs). This recognition activates multiple signaling pathways that lead to the production of interferons (IFNs) and various cytokines, playing a key role in antiviral defense. The IFN family consists of three types – types I, II, and III – which exhibit broad-spectrum antiviral activity [17,18]. IFN-I (IFNα/β), produced through the Toll-like receptor (TLR) pathway, retinoic acid-inducible gene I-like receptor (RLR) pathway, and cyclic GMP-AMP synthase/stimulator of interferon genes (cGAS/STING) pathway, plays a crucial role in the antiviral process. IFNα/β is secreted outside the cell and binds to its receptor subunits, interferon alpha and beta receptor subunit 1 (IFNAR1) and interferon alpha and beta receptor subunit 2 (IFNAR2), activating the Janus kinase 1 (JAK)-STAT signaling pathway. Upon stimulation, STAT1 is recruited to IFNAR2, where its tyrosine 701 site is phosphorylated by JAK1 [19,20]. Phosphorylated STAT1 and signal transducer and activator of transcription 2 (STAT2) recruit interferon regulatory factor 9 (IRF9) to form the interferon-stimulated gene factor 3 (ISGF3) complex, which migrates to the nucleus and binds to the interferon-stimulated response element (ISRE) in the promoter region to induce the transcription and translation of numerous interferon-stimulated genes (ISGs) with antiviral effects [21–23].

HBoV1 proteins have been reported to promote viral replication and immune evasion by modulating molecules in the interferon signaling pathway. Ning et al. demonstrated that the non-structural protein NP1 of HBoV1 directly interacts with Ku70 and RPA70 to enhance viral DNA replication [22]. Liu et al. found that the NS1 and NS1-70 proteins of HBoV1 target p65 and inhibit the activation of TNF-α-mediated NF-κB signaling [24]. Furthermore, Zhang et al. demonstrated that NP1 disrupts the interaction between IRF3 and IFNβ promoter, thereby suppressing IFNβ production [25]. However, due to the lack of suitable cell lines and animal models, as well as the frequent co-infection with other viruses in HBoV1 infections, our understanding of the pathogenic mechanisms of HBoV1 remains limited.

In this study, we conducted a comprehensive analysis of the role of bocavirus-encoded genes in regulating interferon-mediated antiviral responses and examined the effects of bocavirus proteins on mice infected with other respiratory viruses. Our findings provide a foundation for understanding the pathogenic mechanisms and potential treatments for respiratory diseases in children.

## Materials and methods

### Cells, viruses, and mice

HEK293T, Vero, Hep2, and A549 cells were purchased from the American Type Culture Collection (ATCC, Manassas, USA) and cultured in Dulbecco’s modified Eagle’s medium (DMEM) (Gibco, USA) supplemented with 10% fetal bovine serum (FBS) (EallBio, China) at 37°C in a humidified incubator with 5% CO2.

Vesicular stomatitis virus-green fluorescent protein (VSV-GFP), which expresses GFP as a nonstructural protein, was generously provided by Dr. Chunsheng Dong (Soochow University, Suzhou, China) and was propagated in Vero cells. Respiratory syncytial virus L19 strain were propagated in Hep2 cells. Influenza virus H1N1/PR/8/34 was propagated in 10-day-old embryonated eggs (Beijing Laboratory Animal Research Center, Beijing, China). Cells were infected with these viruses at a multiplicity of infection (MOI) of 1, unless otherwise specified.

BALB/c mice were obtained from the Laboratory Animal Center of Soochow University and raised in a specific pathogen-free (SPF) environment. All animals were maintained in a biosafety level 2 (BSL-2) animal facility. Animal experiments were conducted in accordance with the Guide for the Care and Use of Medical Laboratory Animals (Ministry of Health, People’s Republic of China) and were approved by the Institutional Ethics Committee of Soochow University.

### Antibodies and reagents

The following antibodies were used: rabbit anti-DYKDDDDK (AE063, ABclonal), mouse anti-DYKDDDDK (AE005, ABclonal), rabbit anti-HA (3724S, CST), rabbit anti-Myc (16286–1-AP, Proteintech), rabbit anti-ISG15 (A1182, ABclonal), rabbit anti-IFIT1 (23247–1-AP, Proteintech), rabbit anti-CIG5 (A8271, ABclonal), rabbit anti-JAK1 (3332S, CST), rabbit anti-p-JAK1 (abs130626, Absin), rabbit anti-Tyk2 (abs131318a, Absin), rabbit anti-p-Tyk2 (abs139831, Absin), rabbit anti-STAT1 (A12075, ABclonal), rabbit anti-p-STAT1 (9167S, CST), rabbit anti-STAT2 (4595S, CST), rabbit anti-p-STAT2 (88410S, CST), rabbit anti-IRF9 (DF8179, Affinity), mouse anti-GAPDH (M20006S, Abmart), rabbit anti-LMNB1 (12987–1-AP, Proteintech), rabbit anti-VSV-G (M1510-10, HUABIO), rabbit anti-KPNA1 (ab307438, Abcam), mouse anti-RSV (ab94805, Abcam), rabbit anti-H1N1 (11684-R107, Sino Biological), HRP goat anti-mouse IgG (BS12478, Bioworld), HRP goat anti-rabbit IgG (BS13278, Bioworld), Alexa Fluor 488 goat anti-mouse IgG (5230–0391, SeraCare), Alexa Fluor 647 goat anti-rabbit IgG (ab150083, Abcam), mouse anti-GST (300195, ZENBIO), mouse anti-tubulin (66031–1-Ig, Proteintech).

Transfection reagent (40816ES02) was purchased from YEASEN (Shanghai, China). Recombinant human IFNβ (300-02BC) was obtained from PeproTech (Rocky Hill, USA). Anti-Flag (B26102), anti-HA (B26202) and anti-Myc (B26302) magnetic beads were purchased from Bimake (Houston, USA) and GST beads (SA008025) were from Smart-Lifesciences (Changzhou, China). Human IFNβ ELISA KIT (CB10851-Hu) was obtained from COIBO BIO (Shanghai, China).

### Plasmids

The HBoV1 ORFs were amplified from pcDNA3.1(+)-HBoV1 plasmids purchased from sangon (Shanghai, China), and individually subcloned into a eukaryotic expression vector with a Flag tag. Human JAK1, Tyk2, STAT1, STAT2, KPNA1, KPNA2, KPNA3, KPNA4, KPNA6, and KPNB1 ORFs were amplified from RNA extracted from HEK293T cells and then subcloned into separate eukaryotic expression vectors, each containing either a Flag tag, HA tag, or Myc tag. The primer sequences used in this study are listed in Table S1.

### Dual-luciferase reporter (DLR) assays

HEK293T cells were seeded in 96-well plates and co-transfected with 100 ng of viral protein plasmid, 50 ng of IFNβ-luc or ISRE-luc reporter plasmid, and 5 ng of pRL-TK plasmid. At 24 hours post-transfection, cells were either stimulated with IFNβ (400 U/mL) or infected with virus. After stimulation or infection, luciferase activity was measured using a Dual-Luciferase Reporter Assay Kit (YEASEN, China) following cell lysis. All experiments were performed in triplicate, and the results are presented as the mean ± standard deviation (SD) for each representative experiment.

### RNA extraction and quantitative real-time RT-PCR

Total RNA was extracted from cells according to the manufacturer’s instructions using TRIzol reagent (TIANGEN, China). Reverse transcription was performed using the All-In-One 5 × RT MasterMix kit (Abm, China). The resulting cDNA was then mixed with specific RT-PCR primers and SYBR MonAmp™ ChemoHS qPCR Mix (Monad, China). Quantitative real-time RT-PCR was performed for 40 cycles, with the following conditions: 95°C for 10 s, 60°C for 10 s, and 72°C for 30 s. All assays were carried out in triplicate, and the relative mRNA expression levels were normalized to β-actin or GAPDH levels. Primer sequences used for RT-qPCR are listed in Table S2.

### Mass spectrometry

Samples were separated by 10% SDS-PAGE, and the gel bands were excised and digested with trypsin (ThermoFisher Scientific) to generate peptides. The resulting peptides were analyzed by MALDI (EASY-NLC 1200, USA). The data were then matched against Homo sapiens protein databases for identification.

### Confocal immunofluorescence microscopy

HEK293T cells were cultured on 8-well slides and transfected with the indicated plasmids when they reached 60–70% confluence. At 24 hours post-transfection, cells were stimulated with IFNβ (400 U/mL) and subsequently fixed with 4% paraformaldehyde for 10 minutes. The cells were then permeabilized with 0.1% Triton X-100 for 15 minutes and blocked with 5% fetal bovine serum in PBS for 30 minutes at room temperature. After blocking, cells were incubated with specific primary antibodies overnight at 4°C. Following three washes with PBS, cells were incubated with fluorochrome-conjugated secondary antibodies for 45 minutes in the dark. Nuclei were stained with DAPI for 15 minutes. Images were captured using a Nikon A1 confocal microscope (Nikon, Japan).

### Nuclear-cytoplasmic fractionation assay

Nuclear and cytoplasmic proteins were separated using the NE-PER nuclear and cytoplasmic extraction kit (Thermo Scientific) according to the manufacturer’s instructions. The protein level of p-STAT1 was quantified and normalized to the expression of LMNB1 (nuclear marker) or GAPDH (cytoplasmic marker).

### Immunoblotting and immunoprecipitation

HEK293T cells were harvested in cell lysis buffer following transfection and stimulation. The samples were centrifuged at 12,000 rpm for 10 minutes at 4°C to remove cellular debris, and the supernatant was collected. The supernatant was then incubated with anti-Flag, anti-HA, or anti-Myc magnetic beads at 4°C overnight. After three to five washes with lysis buffer, the immunoprecipitates were eluted by boiling in 5 × SDS loading buffer containing β-mercaptoethanol for 10 minutes. The proteins were separated by SDS-PAGE and transferred to polyvinylidene difluoride (PVDF) membranes. Membranes were blocked with 5% nonfat milk for 30 minutes, then incubated with the appropriate primary antibodies, followed by HRP-conjugated goat anti-mouse or anti-rabbit secondary antibodies. Immunoreactive bands were visualized using ECL detection reagent (Tanon, China).

### GST pull-down

HBoV1 NP1 was cloned into the pGEX-6P-2 vector and transformed into BL21 Star (DE3) cells for protein expression. The GST-tagged NP1 protein was then collected and purified using glutathione beads. HEK293T cells were lysed with cell lysis buffer and incubated with the purified NP1-GST protein conjugated to beads at 4°C overnight. After washing three times, the bound proteins were eluted by boiling in 5 × SDS loading buffer for 10 minutes.

### Virus titration

The titers of VSV-GFP in cell-free supernatants were determined using a median tissue culture infective dose (TCID50) assay, as previously described with slight modifications, using Vero cells. Briefly, HEK293T cells were transfected with the indicated plasmids and then infected with VSV-GFP for 12 hours. The culture supernatants containing VSV-GFP were serially diluted with DMEM and added to a monolayer of Vero cells in 96-well plates. The virus titer (TCID50/mL) of VSV-GFP was calculated using the Reed-Muench method. One TCID50/mL was equivalent to 0.69 PFU/mL [26,27].

### Plaque assay

MDCK cells were infected with influenza virus H1N1 for 2 h at 37°C. Cells were then overlaid with DMEM containing 4% FBS and 1% low-melting-point agarose (Promega, USA) and incubated for 4 days. Plates were fixed with 4% formaldehyde and stained with crystal violet to visualize plaques.

### Lentivirus preparation

HEK293T cells were co-transfected with plv-HBoV1 NP1-Flag or control vector (pLVX-puro-Flag) along with two helper plasmids, pVSVG and psPAX2. The culture supernatant containing lentiviruses was collected 48 hours post-transfection and stored at −80°C for subsequent experiments.

### Viral infection in vivo

Twelve 8-week-old female BALB/c mice were randomly divided into two groups (*n* = 6 per group). The control group received tail vein injection of control lentivirus (2 × 10^7^ PFU), while the experimental group was administered NP1 lentivirus (2 × 10^7^ PFU) via identical delivery. Seven days post-injection, all mice in both groups were intranasally inoculated with H1N1 virus (1 × 10^6^ PFU). Four days after viral challenge, mice were euthanized by cervical dislocation and lung tissues were harvested for subsequent analysis. Throughout the experiment, body weight measurements were recorded. Anesthesia was induced using 1.25% tribromoethanol administered intraperitoneally at a dosage of 1.2 mL per 100 g body weight.

### Histological analysis

Lung tissues from both experimental and control mice were fixed in 4% formaldehyde, embedded in paraffin, and sectioned. Paraffin sections were stained with hematoxylin and eosin, and histological changes were observed under a light microscope (Nikon, Tokyo, Japan).

### Statistical analysis

Data were analyzed using Prism 8 software (GraphPad Software, San Diego, USA). Results are expressed as mean ± SD. The significance of differences between two groups was assessed using an unpaired two-tailed Student’s *t*-test, with a significance threshold of *p* < 0.05. Statistical significance is indicated as follows in all figures: *p < 0.05, **p < 0.01, ***p < 0.001, and ****p < 0.0001.All data shown represent three or more biological replicates, unless otherwise stated.

## Results

### HBoV1 VP1, VP2, and NP1 proteins antagonize IFNβ production

**Figure 1.**
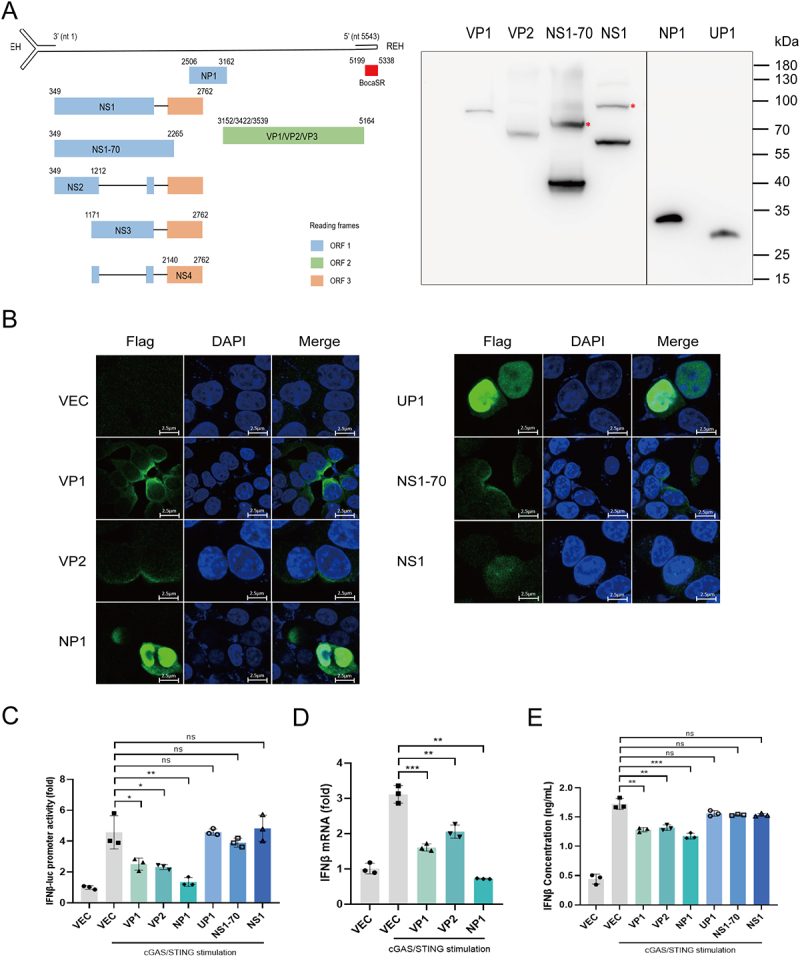
HBoV1 proteins antagonize IFNβ production.

To investigate the relationship between IFN-I and HBoV1 proteins, we cloned six HBoV1 genes into a mammalian overexpression vector, pEGFP-Flag-N1, using homologous recombination based on the viral genome structure (Figure 1(A)). These plasmids were then transfected into HEK293T cells, and Western blotting was performed to confirm their expression. The results indicated that all six viral plasmids were successfully expressed (Figure 1(A)). Additionally, immunofluorescence was used to determine the subcellular localization of the HBoV1 proteins. The findings showed that VP1, VP2, and NS1-70 were predominantly located in the cytoplasm, while UP1 and NS1 were primarily found in the nucleus. NP1 was detected in both the cytoplasm and nucleus (Figure 1(B)).

(A) Genome structure of HBoV1 and expression of HBoV1 proteins. C-terminally Flag-tagged viral proteins were expressed in HEK2933T cells and analyzed by Western blotting using an anti-Flag antibody. (B) HEK293T cells were transfected with C-terminally Flag-tagged viral plasmids (200 ng/well) or an empty vector. Immunofluorescence was performed to determine the localization of HBoV1 proteins in the cells. Scale bar, 2.5 µm. (C) Effect of HBoV1-encoded proteins on IFNβ promoter activity in response to cGAS/STING. HEK293T cells were co-transfected with the Firefly luciferase reporter plasmid pIFNβ-luc, the Renilla luciferase control plasmid pRL-TK, cGAS and STING plasmids, and viral protein-expressing plasmids. Empty plasmids were used as controls. (D) HEK293T cells cultured in 48-well plates were transfected with empty vector (300 ng) or viral protein-expressing plasmids (300 ng), along with cGAS (50 ng) and STING (10 ng) plasmids. After 24 hours, cells were harvested for RNA extraction and subsequent RT-qPCR analysis to assess IFNβ expression. (E) HEK293T cells were transfected with either a control plasmid or individual HBoV1 protein-expressing plasmids and subsequently stimulated with cGAS/STING. Supernatants were collected 24 h post-stimulation, and IFN-β levels were quantified by ELISA. Data are representative of 3 independent experiments and presented as mean ± SD. ns, non-significant; * *p* < 0.05, ** *p* < 0.01, and *** *p* < 0.001 (Student’s t-test).

To investigate the effects of HBoV1 proteins on IFNβ production, we used the cGAS/STING pathway as a model to simulate DNA virus infection. The six viral plasmids encoding various HBoV1 proteins were co-transfected with IFNβ-Luc and pRL-TK into HEK293T cells. After stimulating the cells with cGAS/STING, we measured the impact of HBoV1 proteins on IFNβ promoter activity using dual luciferase reporter (DLR) assays. The results showed that VP1, VP2, and NP1 proteins inhibited IFNβ promoter activity compared to the control group (Figure 1(C)). We then selected these three proteins to assess their effects on IFNβ mRNA levels in cGAS/STING-stimulated cells using RT-qPCR. As shown in Figure 1(D), VP1, VP2, and NP1 proteins significantly reduced IFNβ mRNA levels. Meanwhile, ELISA was performed to quantify IFN-β levels in the supernatants of HEK293T cells transfected with VP1, VP2, or NP1 expression plasmids and stimulated with cGAS/STING. The results showed that all three viral proteins significantly reduced IFN-β production (Figure 1(E)), indicating that HBoV1 VP1, VP2, and NP1 inhibit IFN-β expression.

### HBoV1 VP1, VP2, and NP1 proteins suppress IFN signaling and ISGs production

After the production of IFN-I, it is secreted extracellularly and binds to its receptors (IFNAR1 and IFNAR2) to activate the JAK-STAT signaling pathway. The ISGF3 complex, consisting of p-STAT1, p-STAT2, and IRF9, translocates to the nucleus, where it binds to the ISRE promoter, thereby facilitating the transcription and translation of various ISGs with antiviral effects.

**Figure 2.**
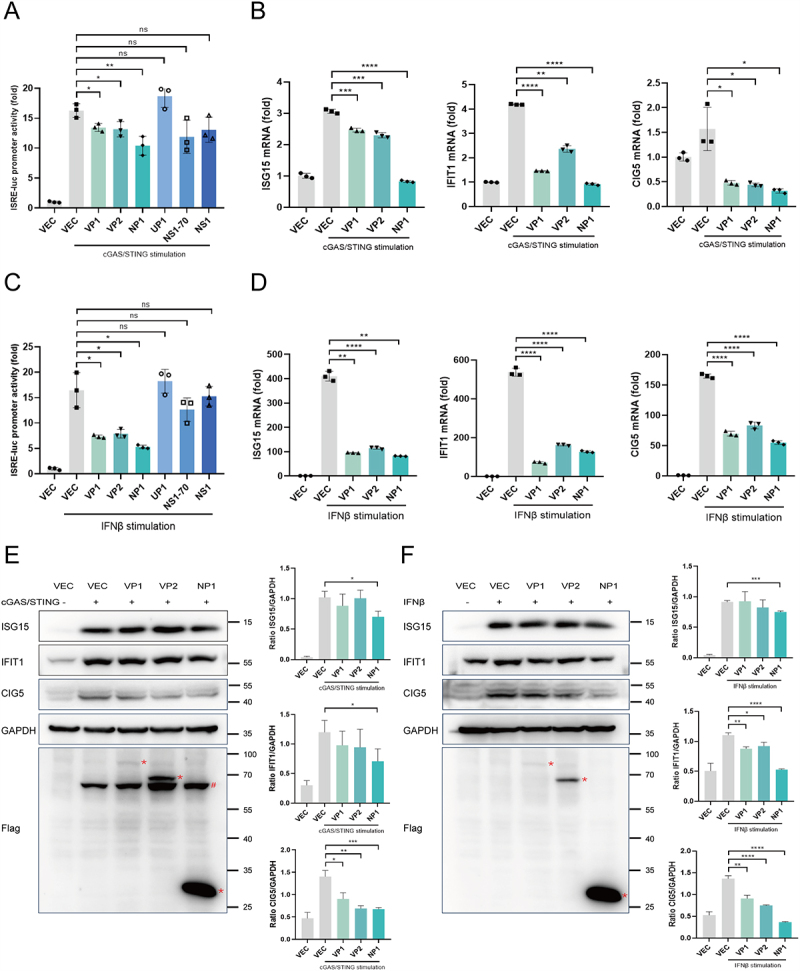
HBoV1 proteins antagonize ISRE and ISG production.

Six HBoV1 plasmids encoding viral proteins were co-transfected with ISRE-Luc and pRL-TK into HEK293T cells. The cells were then stimulated with cGAS/STING. The results showed that VP1, VP2, and NP1 significantly inhibited ISRE promoter activity (Figure 2(A)). To assess the impact of VP1, VP2, and NP1 on ISG mRNA levels (ISG15, IFIT1, CIG5), RT-qPCR was performed following cGAS/STING stimulation. As shown in Figure 2(B), VP1, VP2, and NP1 significantly reduced mRNA levels of ISG15, IFIT1, and CIG5. Additionally, direct stimulation of HEK293T cells with IFNβ (400 U/mL) produced similar results, with VP1, VP2, and NP1 significantly inhibiting both ISRE promoter activity (Figure 2(C)) and mRNA levels of ISG15, IFIT1, and CIG5 (Figure 2(D)). Meanwhile, Western blotting was used to assess the protein levels of ISG15, IFIT1, and CIG5. The results showed that NP1 most significantly suppressed the expression of these proteins following stimulation with either cGAS/STING (Figure 2(E)) or IFN-β (Figure 2(F)).

(A & C) Effect of HBoV1-encoded proteins on ISRE promoter activity in response to cGAS/STING (A) or IFNβ (C) stimulation was analyzed by dual luciferase assays. (B & D) HEK293T cells cultured in 48-well plates were transfected with empty vector (300 ng) or viral protein-expressing plasmids (300 ng) and stimulated with cGAS (50 ng) and STING (10 ng) plasmids (B) or IFNβ (400 U/mL) (D). After 24 hours, cells were harvested for RNA extraction and subsequent RT-qPCR analysis to assess the expression of ISGs (ISG15, IFIT1, and CIG5). (E & F) HEK293T cells cultured in 12-well plates were transfected with empty vector (1000 ng) or viral protein-expressing plasmids (1000 ng) and stimulated with cGAS (150 ng) and STING (30 ng) plasmids (E) or IFNβ (400 U/mL) (F). 24 hours later, cells were harvested for Western blotting to assess the expression of ISGs (ISG15, IFIT1, and CIG5). The gray densities of the indicated bands were analyzed by ImageJ software and normalized to GAPDH. #: cGAS, constructed with an N-terminal FLAG tag, was also detected by anti-Flag antibody in Western blotting. Data are representative of 3 independent experiments and presented as mean ± SD. ns, non-significant; *p < 0.05, **p < 0.01, ***p < 0.001, and ****p < 0.0001 (Student’s t-test).

### NP1 inhibits STAT1’s nuclear import

Previous studies have shown that HBoV1 proteins inhibit IFN-I production by interacting with molecules in the IFN-I signaling pathway, such as RIG-I and IRF3, thereby promoting viral replication or facilitating immune evasion [25,28]. However, our findings reveal a significant gap in understanding the effects of HBoV1 proteins on downstream signaling pathways of IFN-I. In our study, we observed that HBoV1 proteins exert a stronger inhibitory effect on ISRE promoter activity and ISG mRNA levels upon IFNβ stimulation. This observation highlights our growing interest in the complex molecular mechanisms through which HBoV1 proteins may disrupt the IFN-I signaling cascade.

**Figure 3.**
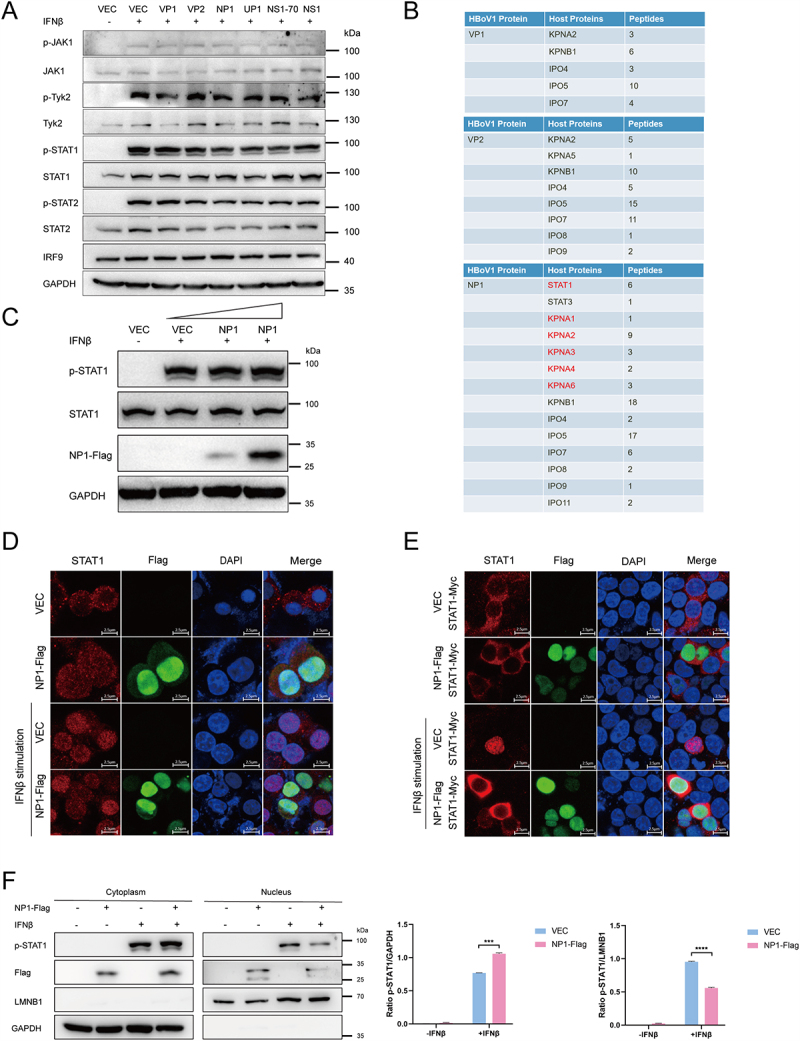
NP1 inhibits STAT1’s nuclear import.

We overexpressed the six HBoV1 protein plasmids in HEK293T cells and stimulated the cells with IFNβ (400 U/mL) for 12 hours after 24 hours of transfection. As shown in Figure 3(A), these six viral proteins did not alter the expression levels of molecules downstream of the IFN-I signaling pathway [JAK1, Tyrosine kinase 2 (Tyk2), STAT1, STAT2, IRF9] or their phosphorylation status (p-JAK1, p-Tyk2, p-STAT1, p-STAT2).

(A) Phosphorylation of JAK1, Tyk2, STAT1, and STAT2. HEK293T cells were transfected with viral protein-encoding plasmids (1.5 μg), treated with IFNβ (400 U/mL) for 12 h, and analyzed by Western blotting. (B) Partial results of the IP-MS assay using Flag-tagged VP1, VP2, or NP1 as bait. HEK293T cells were transfected with plasmids encoding Flag-tagged VP1, VP2, or NP1 proteins. At 24 hours post-transfection (hpt), cells were stimulated with IFNβ (400 U/mL) for 12 h. Co-IP was performed by incubating cell lysates with anti-Flag magnetic beads overnight. Eluted proteins were subjected to Western blotting to confirm successful IP of viral proteins and processed for protein identification by mass spectrometry. (C) Phosphorylation of STAT1. HEK293T cells were transfected with NP1-encoding plasmids at different concentrations (0 ng, 500 ng, 1500 ng), treated with IFNβ (400 U/mL) for 12 h at 24 hpt, and analyzed by Western blotting. (D & E) Nuclear translocation of STAT1. HEK293T cells were transfected with NP1-Flag or empty vector, or co-transfected with STAT1-Myc. At 24 hpt, cells were treated with IFNβ (400 U/mL) for 12 h. Cells were then fixed with 4% paraformaldehyde, permeabilized with 0.1% Triton X-100, and blocked with 2% FBS. Cells were incubated with primary antibodies (anti-Flag and anti-STAT1) and secondary antibodies (anti-Alexa Fluor 488 and anti-Alexa Fluor 647). Scale bar, 2.5 µm. (F) HEK293T cells were transfected with NP1-Flag for 24 h and then stimulated with IFNβ (400 U/mL) for 12 h. p-STAT1 levels in the nucleus and cytoplasm were determined by immunoblotting. GAPDH and LMNB1 served as cytoplasmic and nuclear protein controls, respectively. The gray densities of the indicated bands were analyzed by ImageJ software and normalized to GAPDH or LMNB1.

Given that HBoV1 VP1, VP2, and NP1 exhibited a more pronounced inhibitory effect on IFNβ production and ISG expression, we selected antibodies against VP1, VP2, and NP1 for immunoprecipitation coupled with mass spectrometry (IP-MS). Analysis of the mass spectrometry data revealed that NP1 May interact with STAT1 (Figure 3(B)). To further investigate whether NP1 affects the phosphorylation of STAT1, cells were transfected with the NP1 plasmid at different concentrations (0 ng, 500 ng, 1500 ng) and stimulated with IFNβ (400 U/mL). The results showed that NP1 did not affect the expression or phosphorylation of STAT1 (Figure 3(C)). Therefore, we hypothesized that NP1 might influence STAT1’s nuclear import.

To test this hypothesis, we overexpressed the NP1-Flag plasmid in HEK293T cells, stimulated the cells with IFNβ (400 U/mL), and assessed the intracellular localization of STAT1 by immunofluorescence. As shown in Figure 3(D,E) , NP1 inhibited STAT1 nuclear import. Additionally, a nuclear-cytoplasmic fractionation assay confirmed that p-STAT1 levels were increased in the cytoplasm and decreased in the nucleus in the presence of NP1, indicating that NP1 inhibits STAT1 nuclear translocation (Figure 3(F)).

### NP1 interacts with STAT1 and KPNA1

Cytoplasmic-to-nuclear transport is a crucial process in eukaryotic cells. Molecules entering the nucleus require the assistance of the nuclear transport protein family, particularly importins. Importin-α (KPNA) was the first to be discovered and functions as a linker protein, connecting nuclear import molecules containing classical nuclear localization signals (cNLS) with importin-β (KPNB). In humans, there are seven types of KPNAs: KPNA1 (importin-α5), KPNA2 (importin-α1), KPNA3 (importin-α4), KPNA4 (importin-α3), KPNA5 (importin-α6), KPNA6 (importin-α7), and KPNA7 (importin-α8) [29,30].

**Figure 4.**
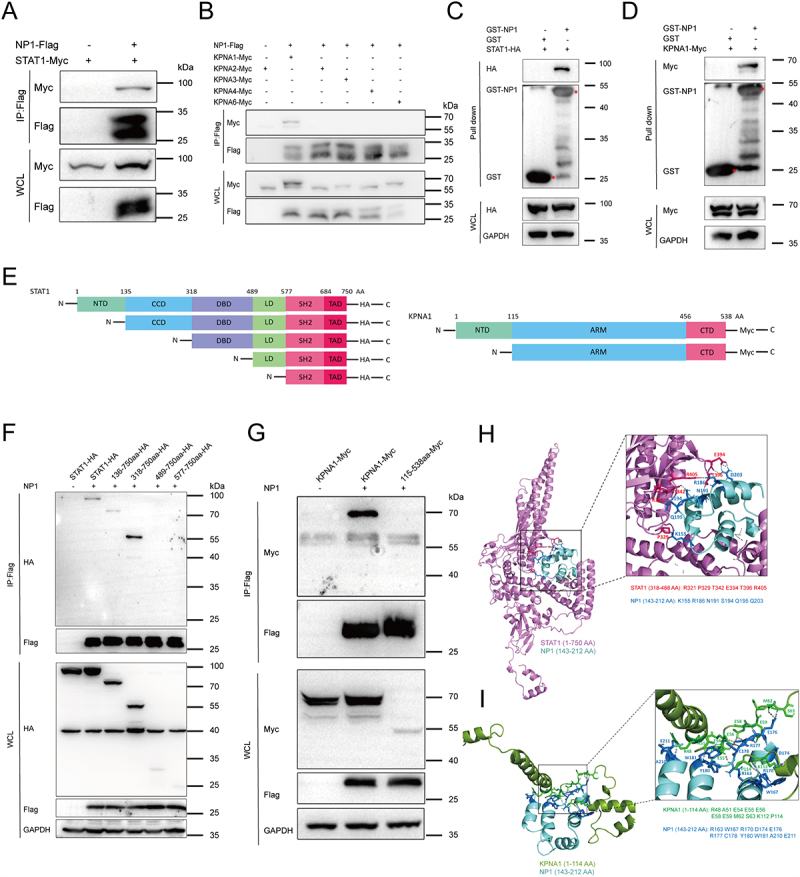
NP1 interacts with STAT1 and KPNA1.

We have demonstrated that NP1 inhibits the nuclear translocation of STAT1. But how does this process occur? The nuclear translocation of the ISGF3 complex, consisting of p-STAT1, p-STAT2, and IRF9, is mediated by the nuclear transport protein family. As shown in Figure 3(B), NP1 May interact with STAT1 and members of the nuclear transport protein family, including KPNA1, KPNA2, KPNA3, KPNA4, and KPNA6. To confirm this, we co-transfected NP1-Flag with STAT1-Myc and these nuclear transport proteins in HEK293T cells and verified the interactions between NP1-STAT1 and NP1-KPNA1 using co-immunoprecipitation Figure 4(A,B). *In vitro*, GST protein and GST-NP1 protein were purified, and as shown in Figure 4(C,D), NP1 directly interacted with STAT1 and KPNA1 in a GST pull-down assay.

(A & B) Co-IP of NP1 with STAT1 or KPNA proteins. HEK293T cells were co-transfected with NP1-Flag and STAT1-Myc plasmids or KPNA-Myc plasmids. At 24 hpt, Co-IP was performed by incubating cell lysates with anti-Flag magnetic beads overnight. After extensive washing, the eluate was analyzed by Western blotting. (C & D) Direct interaction between NP1 and STAT1 or KPNA1. HBoV1 NP1 was cloned into pGEX-6P-2 and transformed into BL21 Star (DE3) for protein expression. GST-tagged NP1 protein was collected and purified using glutathione beads. HEK293T cells were lysed in cell lysis buffer and incubated with purified NP1 protein conjugated to beads at 4°C overnight. After extensive washing, the eluate was analyzed by Western blotting with the indicated antibodies. (E) Schematic representation of protein domains of STAT1 and KPNA1. (F & G) Co-IP of NP1 with domains of STAT1 or KPNA1. (H & I) Molecular docking. PyMOLWin software was used to simulate the interactions between NP1 and STAT1 or NP1 and KPNA1.

Based on the domain structure of STAT1 and KPNA1, truncations of these proteins were constructed through homologous recombination (Figure 4(E)). Co-transfection of the truncations with NP1 revealed that NP1 interacts with the 318–488 AA region of STAT1 and the 1–114 AA region of KPNA1, as demonstrated by immunoprecipitation (Figure 4(F,G)). The 318–488 AA region of STAT1 corresponds to its DBD, while the 1–114 AA region of KPNA1 includes the crucial IBB domain. Additionally, the PDB files for NP1, STAT1, and KPNA1 were retrieved from the SWISS-MODEL (https://swissmodel.expasy.org/) and RCSB PDB (https://www.rcsb.org/) websites. Molecular docking for the NP1-STAT1 and NP1-KPNA1 complexes was performed using ClusPro (https://cluspro.org/). Subsequently, the docking results were visualized with PyMOLWin software. The results indicate the presence of interactions between NP1 (K155, R186, N191, S194, Q195, D203) and STAT1 (R321, P329, T342, E394, T396, R405), as well as between NP1 (R163, W167, R170, D174, E176, R177, C178, Y180, W181, A210, E211) and KPNA1 (R48, A51, E54, E55, E56, E58, E59, M62, S63, K112, P114) (Figure 4(H,I)). These results suggest that NP1 interacts with the 318–488 AA region of STAT1 (DBD) and the 1–114 AA region of KPNA1, which contains the IBB domain.

### NP1 inhibits the interaction between STAT1-KPNA1 and KPNA1-KPNB1

KPNA1 is the primary nuclear transport protein responsible for mediating the nuclear translocation of p-STAT1. Upon phosphorylation, the DBD of STAT1 is exposed, allowing it to bind to KPNA1 [31,32]. Concurrently, KPNB1 interacts with the IBB domain of KPNA1, forming a trimeric complex of p-STAT1-KPNA1-KPNB1 that translocates into the nucleus through the nuclear pore complex. Once inside the nucleus, RanGTP binds to KPNB1, causing the dissociation of the ternary complex and releasing p-STAT1 into the nuclear environment [32,33].

**Figure 5.**
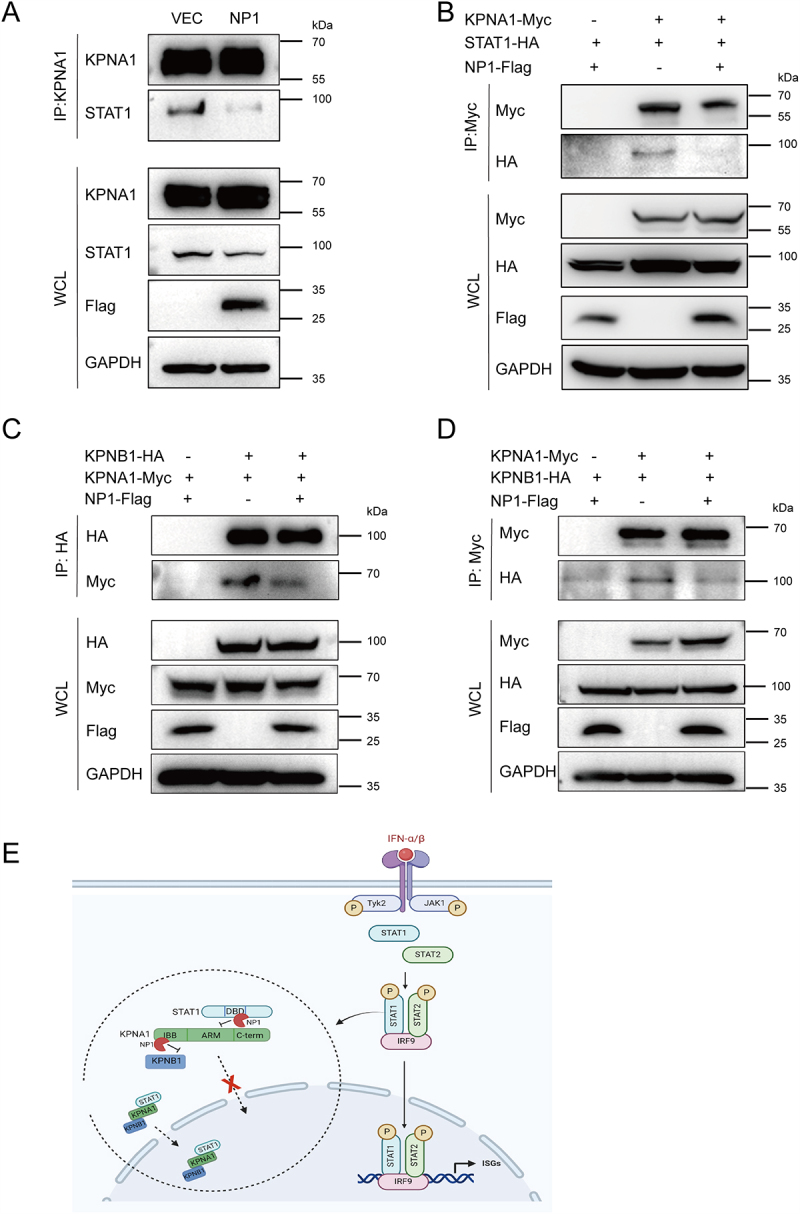
NP1 inhibits the interaction between STAT1-KPNA1 and KPNA1-KPNB1.

To explore the role of NP1 in modulating the interaction between STAT1 and its nuclear import proteins, we conducted immunoprecipitation experiments using HEK293T cells overexpressing NP1. Our results revealed that overexpression of NP1 significantly inhibited the interaction between STAT1 and KPNA1, as shown in (Figure 5(A,B)). This suggests that NP1 acts as a negative regulator within the STAT1-KPNA1 axis. Based on the data in Figure 4(F), we propose that NP1 competes with KPNA1 for binding to the DBD of STAT1, thereby antagonizing the formation of the STAT1-KPNA1 complex and inhibiting STAT1’s nuclear import.

(A & B) HEK293T cells were co-transfected with or without NP1-Flag plasmid. Co-IP assays were performed using anti-KPNA1 or anti-Myc antibodies. (C & D) HEK293T cells were transfected with plasmids encoding Myc-tagged KPNA1 and HA-tagged KPNB1, with or without NP1-Flag plasmid. Co-IP assays were performed using anti-HA or anti-Myc antibodies. (E) Mechanistic diagram illustrating how NP1 inhibits the nuclear import of STAT1.

KPNB1-HA plasmid was constructed through homologous recombination, as KPNB1 is involved in the nuclear import of STAT1 and was identified in our previous IP-MS experiments. We confirmed that NP1 inhibits the interaction between KPNA1 and KPNB1 using immunoprecipitation (Figure 5(C,D)). Based on the data in Figure 4(G), we conclude that NP1 competes for the binding of the 1–114 AA region of KPNA1, which contains the IBB domain, thereby inhibiting the interaction between KPNA1 and KPNB1 and preventing STAT1’s nuclear import.

These findings suggest that NP1 disrupts the formation of the STAT1/KPNA1/KPNB1 complex by interacting with the DBD of STAT1 and the N-terminal IBB domain of KPNA1. This disruption impairs STAT1 nuclear transport, suppresses ISRE promoter activity, reduces ISG expression, and facilitates immune evasion (Figure 5(E)).

### NP1 promotes the replication of other viruses

**Figure 6.**
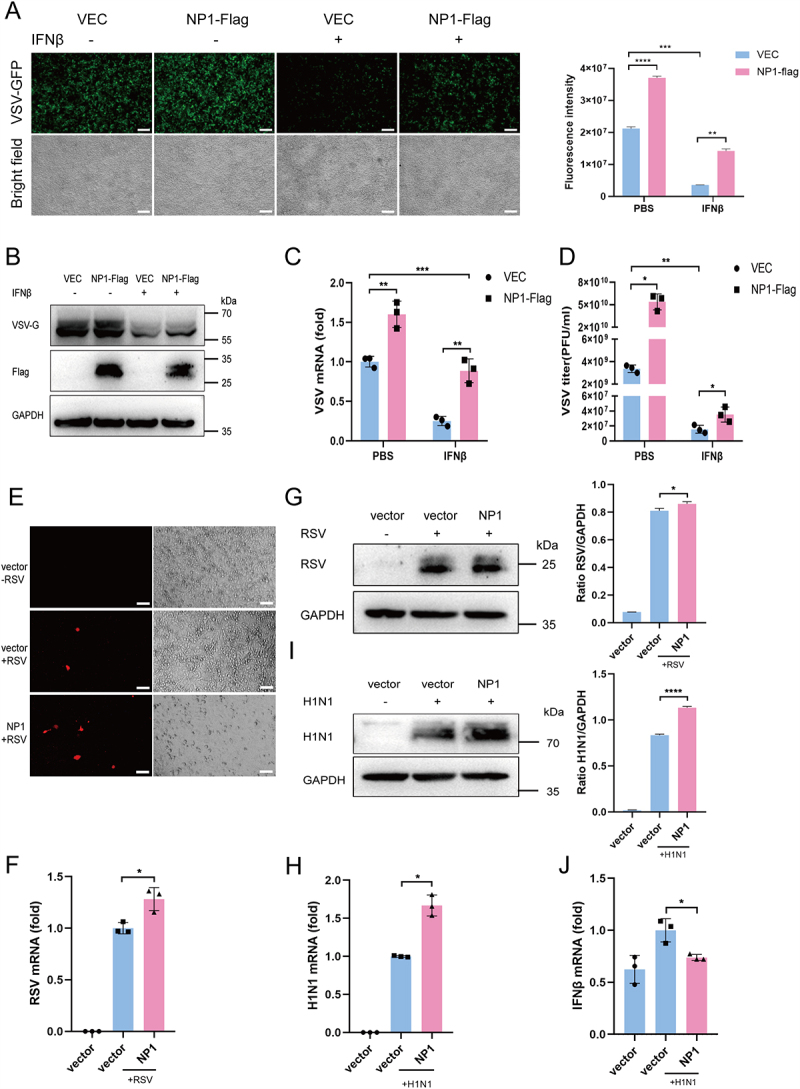
NP1 promotes the replication of co-infected viruses.

The essential role of NP1 in the viral replication process has been well established. Our findings further demonstrate that NP1 inhibits the promoter activity of IFNβ and ISRE, as well as the mRNA levels of IFNβ, ISG15, IFIT1, and CIG5 (Figure 1 and Figure 2). To assess the impact of NP1 on viral replication, HEK293T cells were infected with vesicular stomatitis virus (VSV) (MOI = 0.5), a well-known model virus, for 8 hours. Viral content was evaluated at both protein and mRNA levels using fluorescence microscopy (Figure 6(A)), Western blotting (Figure 6(B)), RT-qPCR (Figure 6(C)), and the TCID50 assay (Figure 6(D)). The results revealed that NP1 effectively suppresses the antiviral effects of IFNβ, thereby promoting VSV-GFP replication.

(A-D) HBoV1 NP1 protein facilitates VSV replication. HEK293T cells were transfected with the indicated plasmids. At 24 hpt, cells were treated with IFNβ (400 U/mL) for 12 h. GFP-positive cells were visualized by confocal microscopy (A). The replication of VSV was assessed by Western blotting using VSV-G antibody (B). RT-qPCR was performed to measure VSV mRNA replication levels (C). TCID50 assay was used to determine the titers of extracellular VSV-GFP (PFU/mL) (D). Scale bar, 50 µm. (E-G) NP1 protein facilitates the replication of RSV. Hep2 cells were transfected with the indicated plasmids. At 24 hpt, cells were infected with RSV for 48 hours. RFP-positive cells were visualized by confocal microscopy (E). Scale bar, 50 µm. RT-qPCR was performed to measure RSV mRNA replication levels (F). The replication of RSV was assessed by Western blotting using RSV antibody The gray densities of the indicated bands were analyzed by ImageJ software and normalized to GAPDH (G). (H-J) NP1 protein facilitates the replication of H1N1. A549 cells were transfected with the indicated plasmids. At 24 hpt, cells were infected with H1N1 for 48 hours. RT-qPCR was performed to measure H1N1 mRNA replication levels (H). The replication of H1N1 was assessed by Western blotting using H1N1 antibody. The gray densities of the indicated bands were analyzed by ImageJ software and normalized to GAPDH (I). The mRNA level of IFNβ was determined by RT-qPCR (J). Data are representative of 3 independent experiments and presented as mean ± SD. *p < 0.05, **p < 0.01, ***p < 0.001, and ****p < 0.0001 (Student’s t-test).

NP1 was overexpressed in Hep2 cells, followed by infection with RSV 24 hours later. Forty-eight hours post-infection, RSV content was assessed using virus fluorescence imaging (Figure 6(E)), RT-qPCR analysis (Figure 6(F)), and Western blotting (Figure 6(G)). Our findings indicate that NP1 facilitates RSV replication. Similarly, when NP1 was overexpressed in A549 cells and the cells were reinfected with H1N1 after 24 hours, enhanced H1N1 replication was observed, as demonstrated by RT-qPCR analysis (Figure 6(H)) and Western blotting (Figure 6(I)). Additionally, NP1-mediated inhibition of IFNβ production was evident from the reduced mRNA levels of IFNβ (Figure 6(J)), suggesting that HBoV1 promotes H1N1 replication by partially inhibiting IFNβ production.

### NP1 promotes H1N1 replication in vivo

**Figure 7.**
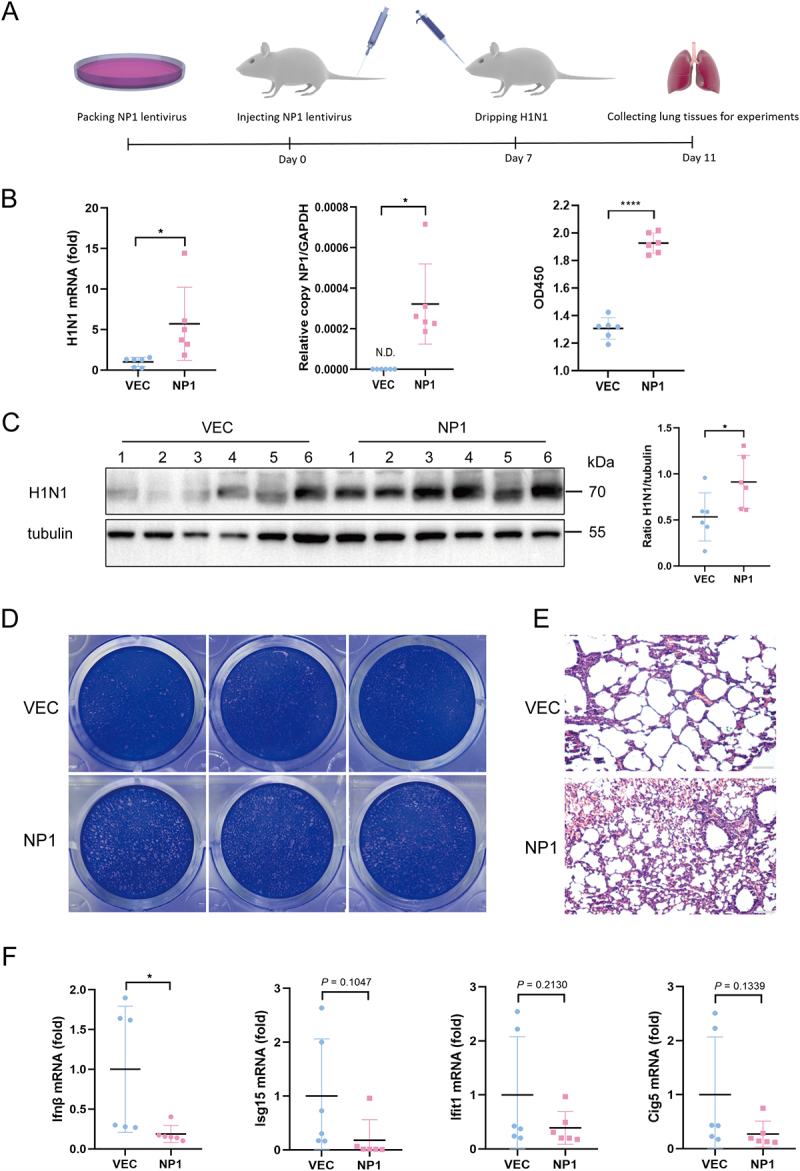
NP1 promotes H1N1 replication in mice.

HBoV1 exclusively infects highly differentiated or polarized primary human airway epithelial cells, which require specialized air-liquid interface culture techniques. As a result, the absence of suitable cell lines and animal models for viral culture and experimentation presents significant challenges in studying HBoV1. To investigate the role of HBoV1 NP1, we utilized plv-NP1-Flag lentivirus for packaging and subsequently infected mice via tail vein injection (2 × 10^7^ PFU). Seven days post-injection, the mice were intranasally instilled with H1N1 (1 × 10^6^ PFU). Four days after instillation, the mice were sacrificed, and lung tissues were collected for further analysis (Figure 7(A)). RT-qPCR, Western blotting, and plaque assays were performed to assess H1N1 viral load (Figure 7(B-D)). The results showed that mice pre-infected with NP1-expressing lentivirus exhibited increased H1N1 mRNA and protein levels, as well as higher viral titers, following co-infection with H1N1 *in vivo*. Additionally, HE staining of lung tissue samples revealed more severe lesions in the lungs of mice co-infected with NP1 lentivirus and H1N1, characterized by disordered tissue structure, thickened septa, and significant infiltration of inflammatory cells (Figure 7(E)). These findings suggest that NP1 co-infection enhances H1N1 replication. Furthermore, RT-qPCR analysis of lung tissue samples from co-infected mice showed significantly lower Ifnβ mRNA levels compared to those infected with H1N1 alone. Additionally, Isg15, Ifit1, and Cig5 levels were also lower in the co-infected lungs, although these differences did not reach statistical significance (Figure 7(F)). These results indicate that NP1 promotes H1N1 replication by inhibiting IFNβ production and mRNA levels of certain ISGs.

(A) Flowchart of BALB/c mouse infection with NP1 lentivirus (2 × 10⁷ PFU) and H1N1 (1 × 106 PFU). Mice co-infected with both NP1 lentivirus and H1N1 served as the experimental group, while mice infected only with H1N1 served as the control group. (B) Analysis of viral mRNA from lung tissues. Results were normalized to mouse GAPDH expression. Each dot represents data from an individual mouse. RT-qPCR and ELISA assays were employed to verify the efficacy of lenti-NP1. (C) Immunoblot analysis of H1N1 in lung tissues. Each band represents a tissue sample from one mouse The gray densities of the indicated bands were analyzed by ImageJ software and normalized to tubulin. (D) Plaque assays were performed using lysates collected from lung tissue on MDCK cells. (E) Micrographs of hematoxylin and eosin-stained lung sections from mice. Scale bar, 100 µm. (F) RT-qPCR analysis of Ifnβ, Isg15, Ifit1, and Cig5 mRNA levels in lung samples from infected mice. The relative mean expression levels of genes in the control group were set to “1.” Representative results from three independent experiments. *p < 0.05 and ****p < 0.0001 (Student’s t-test).

## Discussion

Respiratory infections are among the most common diseases in children and the leading cause of death in children under the age of 5 worldwide. HBoV, a relatively recent addition to the group of respiratory viruses discovered over the past two decades, has garnered increasing attention for its association with pediatric respiratory diseases and their disease burden. HBoV1, in particular, is primarily linked to respiratory infections, as it can only infect highly differentiated or polarized primary human respiratory epithelial cells, requiring specialized culture methods involving an air-liquid interface. Consequently, there is a scarcity of suitable cell lines and animal models for HBoV1 culture and experimentation, and research on the virus’s pathogenesis remains limited. In this study, we focused on the role of HBoV1-encoded proteins in modulating innate immunity. Specifically, we found that the HBoV1 NP1 protein inhibits the production of IFNβ, a finding consistent with the work of Zhang et al. [25]. Moreover, NP1 interferes with the formation of the STAT1/KPNA1/KPNB1 complex by binding to the DBD of STAT1 and the N-terminal region of KPNA1, thereby competitively inhibiting the nuclear import of STAT1 and suppressing the expression of various ISGs.

It has been reported that the N-terminal region of HBoV1 NP1, spanning residues 7 to 50, contains a non-classical nuclear localization signal (ncNLS) and is primarily localized in the nucleus [34,35]. However, intriguingly, we observed NP1 in both the cytoplasm and nucleus during immunofluorescence and nuclear fractionation experiments. To explain this phenomenon, we propose that the receptor responsible for HBoV1 entry into cells remains unidentified. Given that parvoviruses are known to enter cells through receptor-mediated endocytosis, followed by transport via early to late endosomes before reaching the nucleus [36,37], it is likely that HBoV1 also follows a similar pathway. This suggests that NP1’s cytoplasmic localization may result from the early stages of viral entry and trafficking, while its nuclear presence could reflect its role in the virus’s life cycle after it has entered the nucleus.

Upon nuclear translocation, the viral genome engages host DNA damage and repair machinery for recognition. Complementary strand synthesis of the single-stranded DNA (ssDNA) is initiated through host enzymatic activity, generating transcriptionally active double-stranded intermediates. This process drives sequential expression of non-structural (NS) proteins, which orchestrate viral genome replication and coordinate packaging into progeny virions [11]. The double-stranded DNA (dsDNA) template is transcribed to produce viral proteins and the long non-coding RNA, BocaSR, while DNA replication occurs. The capsid proteins, produced in the cytoplasm, form oligomers before being transported to the nucleus, where they assemble into mature viral capsids [38,39]. Finally, the fully assembled virus particles are released from the nucleus into the cytoplasm, and from there, they are transported to infect new cells [11]. The phenomenon we have observed may also reflect the process by which NP1 is synthesized on ribosomes and subsequently enters the nucleus. Based on our study of the mechanism by which NP1 inhibits STAT1 nuclear import, we hypothesize that NP1 interferes with the formation or translocation of the STAT1/KPNA1/KPNB1 trimer complex during its own nuclear import process. This inhibition likely occurs in the cytoplasm or at the vicinity of the nuclear membrane. However, the precise location of this mechanism requires further investigation to confirm.

Clinical cases have shown that HBoV infections are frequently mixed with other viral and bacterial respiratory or gastrointestinal pathogens, including RSV, influenza virus, human rhinovirus, adenovirus, norovirus, and rotavirus [5–7]. In respiratory samples, up to 83% of infections are mixed, with RSV being the most common co-infection, observed in 89.5% of cases [8,9]. Recently, there has been an increasing number of reports highlighting severe infections caused by HBoV, some of which are life-threatening. However, current studies present conflicting views on whether mixed infections with HBoV exacerbate disease severity.

Some studies have suggested that co-infection of HBoV with other pathogens may exacerbate disease severity [40–42], while others have argued the opposite [43–45]. In both *in vivo* and *in vitro* experiments, we have found that HBoV1 NP1 enhances the replication of H1N1. However, cell lines or animal models suitable for the culture and experimentation of live HBoV1 virus remain severely limited, making it challenging to obtain and culture HBoV1 for use in co-infection experimental models. It has been reported that infectious clones formed by dual genomes of HBoV1 can replicate in HEK293T cells and produce high titers of progeny viral particles upon transfection [46], overcoming the challenges of culturing and obtaining HBoV1. Building on this, we used homologous recombination to generate a full-length clone of HBoV1, named T boca, which partially mimics the expression of all HBoV1 proteins, and confirmed that it also promotes the replication of H1N1 and RSV (Fig. S1 A-G). These findings provide a theoretical basis for understanding co-infections in clinical settings.

Due to the high transfection efficiency of HEK293T cells and the finding that infectious clones formed by dual genomes of HBoV1 can replicate in HEK293T cells and produce high-titer progeny virus particles through transfection [46], we selected this cell line to overexpress individual HBoV1 protein plasmids or to construct a full-length HBoV1 genomic plasmid (T boca). However, HEK293T cells inherently lack a fully functional native cGAS-STING pathway. Although they express low levels of STING protein, they lack functional cGAS enzymatic activity. As cGAS is the key molecule responsible for recognizing cytosolic DNA and synthesizing the second messenger 2’3’-cGAMP, HEK293T cells cannot efficiently produce cGAMP and are therefore insufficient to mount a robust cellular response to DNA or DNA viruses [47,48]. Despite this innate deficiency, HEK293T cells are widely utilized for *in vitro* reconstitution studies of this pathway owing to their high transfection efficiency and ease of genetic modification. For instance, Wu et al. co-transfected human cGAS and STING plasmids into HEK293T cells, confirming that exogenous cGAS can synthesize cGAMP and activate STING-dependent IRF3 phosphorylation and IFN-β production [47]. Accordingly, we also activated this pathway in HEK293T cells by exogenously co-transfecting cGAS and STING plasmids.

In summary, our study demonstrates that HBoV1 NP1 binds to the DBD of STAT1 and the N-terminal region of KPNA1, inhibiting the formation of the STAT1/KPNA1/KPNB1 complex and competitively suppressing the nuclear import of STAT1. These findings provide theoretical support for strategies to control HBoV1 infections in children and offer a foundation for the future development of vaccines and therapeutic interventions.

## Supplementary Material

Supplementary Table S1 and S2.docx

supplementary figure 1.tif

## Date availability statement

All relevant data generated during this study are included in the main paper and Supplementary Information. The raw data has been uploaded to the database (Figshare). The DOI number is 10.6084/m9.figshare.30001363 [49].
