# Supplementary material for: Human bocavirus NP1 antagonizes host type I interferon response through repressing the nuclear transport of STAT1
Source: Virulence. 2025 Oct 15;16(1):2570000. doi: 10.1080/21505594.2025.2570000 (PMC12533952; doi:10.1080/21505594.2025.2570000)
Supplement: Supplementary Table S1 and S2.docx [file KVIR_A_2570000_SM2650.docx]

**Supplementary Table S1** Oligo primers used for plasmid construction in the study

| Primer*^a^* | Sequence*^b^* (5’-3’) |
| --- | --- |
| HBoV1-VP1-F | GCTTCGAATTCTGCAGTCGACACCATGGATTACAAGGATGACGACGATAAGGGTTCTATGCCTCCAATTAAGAGACAGCC |
| HBoV1-VP1-R | ATGGTGGCGACCGGTGGATCCCGCAACACTTTATTGATGTTTGTTTTTACTG |
| HBoV1-VP2-F | GCTTCGAATTCTGCAGTCGACACCATGGATTACAAGGATGACGACGATAAGGGTTCTATGTCTGACACTGACATTCAAGACCA |
| HBoV1-VP2-R | ATGGTGGCGACCGGTGGATCCCGCAACACTTTATTGATGTTTGTTTTTACTG |
| HBoV1-NP1-F | GCTTCGAATTCTGCAGTCGACACCATGAGCTCAGGGAATATGAAAGACA |
| HBoV1-NP1-R | ATGGTGGCGACCGGTGGATCCCGATTGGAGGCATCTGCTTCCA |
| HBoV1-UP1-F | GCTTCGAATTCTGCAGTCGACACCATGTACACTGATCGCGCAAG |
| HBoV1-NS1-UP1-F | CACAGGAGACATCACACCAAGTGACTTGGGGGATTCGGAC |
| HBoV1-NS1-UP1-R | GTCCGAATCCCCCAAGTCACTTGGTGTGATGTCTCCTGTG |
| HBoV1-NS1-R | ATGGTGGCGACCGGTGGATCCCGTTGTCTTTTTTCCCCGATGTAC |
| HBoV1-NS1-70-F | GCTTCGAATTCTGCAGTCGACACCATGGATTACAAGGATGACGACGATAAGGGTTCTATGGCTTTCAATCCTCCTGTGA |
| HBoV1-NS1-70-R | ATGGTGGCGACCGGTGGATCCCGCTTACTTGGTGTGATGTCTCCTGTG |
| STAT1-F | GCTTCGAATTCTGCAGTCGACACCATGTCTCAGTGGTACGAACTTCAG |
| STAT1-R | ATGGTGGCGACCGGTGGATCCCGTACTGTGTTCATCATACTGTCGAAT |
| STAT1 136-750 aa-F | GCTTCGAATTCTGCAGTCGACACCATGTTAGACAAACAGAAAGAGCTTG |
| STAT1 318-750 aa-F | GCTTCGAATTCTGCAGTCGACACCATGGTGGTGGAAAGACAGCC |
| STAT1 489-750 aa-F | GCTTCGAATTCTGCAGTCGACACCATGACTCCACCATGTGCACG |
| STAT1 577-750 aa-F | GCTTCGAATTCTGCAGTCGACACCATGTGCATCATGGGCTTCA |
| KPNA1-F | GCTTCGAATTCTGCAGTCGACACCGCACTGCGAACGCCGGCTG |
| KPNA1-R | ATGGTGGCGACCGGTGGATCCCGTTTTCTTTTATCAAGCACCCT |
| KPNA1 115-538 aa-F | TTGGTACCGAGCTCGGATCCATGAACCCTCCTATTGATGAAGT |
| KPNA1 115-538 aa-R | GCTGGATATCTGCAGAATTCAAGCTGGAAACCTTCCATAGG |
| KPNA2-F | GCTTCGAATTCTGCAGTCGACACCGTTGACTAGGCCTCGGGG |
| KPNA2-R | ATGGTGGCGACCGGTGGATCCCGTGAAGTCAAGAAAAGGGTGGA |
| KPNA3-F | GCTTCGAATTCTGCAGTCGACACCAGTCGGCCCGCGCCTCCCCC |
| KPNA3-R | ATGGTGGCGACCGGTGGATCCCGTTTTATCATATTTGATA |
| KPNA4-F | GCTTCGAATTCTGCAGTCGACACCAGATCGAGGCTGCCTCC |
| KPNA4-R | ATGGTGGCGACCGGTGGATCCCGTCATTATCACAAGCATTTATT |
| KPNA6-F | GCTTCGAATTCTGCAGTCGACACCATATTGTCTACTGAAAGC |
| KPNA6-R | ATGGTGGCGACCGGTGGATCCCGAACTTTGCAACAGGTTTTTATT |
| KPNB1-F | GCTTCGAATTCTGCAGTCGACACCATGGAGCTGATCACCATTCTC |
| KPNB1-R | ATGGTGGCGACCGGTGGATCCCGAGCTTGGTTCTTCAGTTTCCTC |

*^a^*F, forward; R, reverse.

**Supplementary Table S2** Oligo primers used for RT-qPCR analysis of the study

| Primer*^a^* | Sequence (5’-3’) |
| --- | --- |
| Human actin-F | TGACGTGGACATCCGCAAAG |
| Human actin-R | CTGGAAGGTGGACAGCGAGG |
| Human IFNβ-F | CATTACCTGAAGGCCAAGGA |
| Human IFNβ-R | CAGCATCTGCTGGTTGAAGA |
| Human ISG15-F | AGGACAGGGTCCCCCTTGCC |
| Human ISG15-R | CCTCCAGCCCGCTCACTTGC |
| Human IFIT1-F | TGACTCTTTTGCCTCTTTCTTCTAA |
| Human IFIT1-R | TTCTTGGGGTGCTCTGTGG |
| Human CIG5-F | TGGGTGCTTACACCTGCTG |
| Human CIG5-R | GAAGTGATAGTTGACGCTGGTT |
| Mouse Ifnβ-F | TGCGTTCCTGCTGTGCTT |
| Mouse Ifnβ-R | CGTCATCTCCATAGGGATCTTG |
| Mouse Isg15-F | CAGTGCTCCAGGACGGTCTTACC |
| Mouse Isg15-R | CCCGCTGGGACACCTTCTTC |
| Mouse Ifit1-F | GTGGCTCACATAGAGCAGGA |
| Mouse Ifit1-R | AGTTTCCTCCAAGCAAAGGA |
| Mouse Cig5-F | TGCTGGCTGAGAATAGCATTAGG |
| Mouse Cig5-R | GCTGAGTGCTGTTCCCATCT |
| Mouse GAPDH-F | GGCCTTCCGTGTTCCTACC |
| Mouse GAPDH-R | AGCCCAAGATGCCCTTCAGT |
| VSV-F | ACGGCGTACTTCCAGATGG |
| VSV-R | CTCGGTTCAAGATCCAGGT |
| RSV-F | TGCAGTGCAGTTAGCAAAGG |
| RSV-R | GATTGTTTGCTGCTGGTGTG |
| H1N1-F | TTCTAACCGAGGTCGAAACG |
| H1N1-R | ACAAAGCGTCTACGCTGCAG |
| HBoV1 NP1-F | AGACAAGCATCGCTCCTACA |
| HBoV1 NP1-R | TGGCTGATTGGGTGTTCC |

*^a^*F, forward; R, reverse.
