# Supplementary figures and images for: Human bocavirus NP1 antagonizes host type I interferon response through repressing the nuclear transport of STAT1
Source: Virulence. 2025 Oct 15;16(1):2570000. doi: 10.1080/21505594.2025.2570000 (PMC12533952; doi:10.1080/21505594.2025.2570000)

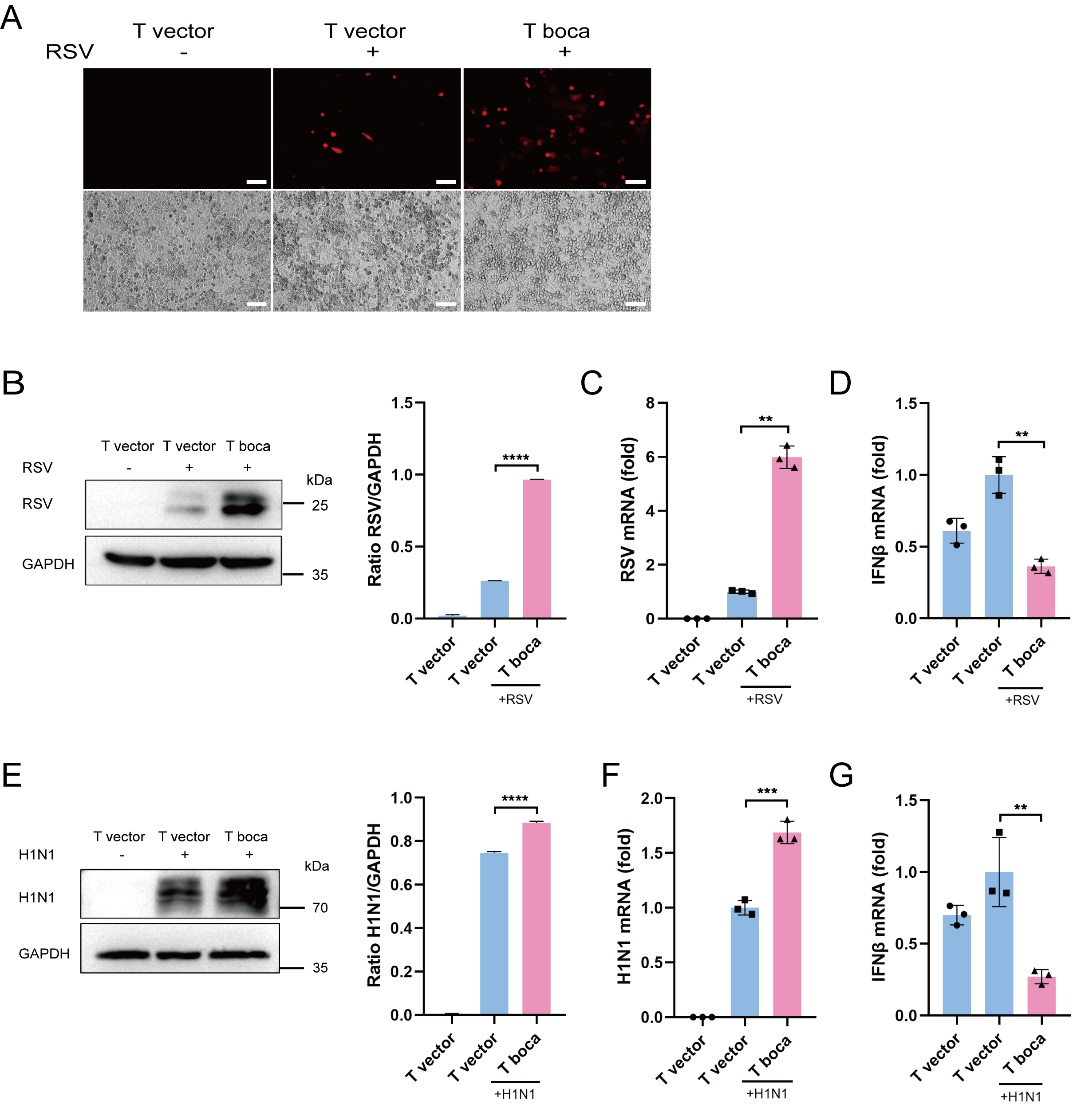

Supplement: supplementary figure 1.tif [file KVIR_A_2570000_SM2649.tif]
